# Leadless Pacemaker Implantation in the Presence of the Bioprosthetic Tricuspid Valve: Case Presentation and Literature Review

**DOI:** 10.1002/ccr3.70077

**Published:** 2025-01-06

**Authors:** Mahsa Mohammadi, MohammadReza Iranian, Sedigheh Saedi, Yaser Toloueitabar, Amirfarjam Fazelifar, Majid Haghjoo

**Affiliations:** ^1^ Rajaie Cardiovascular Medical and Research Institute Tehran Iran; ^2^ Department of Electrophysiology Rajaie Cardiovascular Medical and Research Institute Tehran Iran; ^3^ Cardiac Electrophysiology Research Center Rajaie Cardiovascular Medical and Research Institute Tehran Iran

**Keywords:** bioprosthetic valve, cardiovascular disease, electrophysiology, leadless pacemaker, Micra, tricuspid valve

## Abstract

A 21‐year‐old man, known case of the repaired congenital heart disease, developed complete atrioventricular block (AVB) one week after simultaneous bioprosthetic pulmonary and tricuspid valve replacement and atrial septal defect repair. Considering the persistence of the AVB, it was decided to implant a permanent pacemaker. After considering all available options and the issues related to the patient, it was decided to implant a leadless pacemaker (LLP). A Micra pacemaker was implanted successfully, and the patient was discharged in good condition and without any complications. Follow‐up evaluation showed appropriate LLP and bioprosthetic valve functioning. Limited prior experiences and the present report showed that LLP appears to be an ideal option in the patients with bioprosthetic tricuspid valve complicated by conduction disorders.

**Summary**

- Leadless pacemaker (LLP) is a new technology validated in real‐world setting with advantages of overcoming some limits of the conventional pacing such as infection, lead malfunction, and lead‐related tricuspid valve regurgitation.
- Our case shows LLP is a safe option in patients developing conduction disorders after Bioprosthetic Tricuspid valve replacement.

## 1 Introduction

Postoperative atrioventricular block (AVB) has been reported in 1%–6% of patients after cardiac surgery and 25%–60% of these patients will finally need a permanent pacemaker (PPM) [1, 2, 3, 4]. To avoid tricuspid valve (TV) malfunction, implantation of conventional pacing leads is generally not preferred in the presence of the tricuspid bioprosthesis **[** 5 **]**.

Leadless pacemakers (LLP) have recently become popular in treatment of heart blocks and bradyarrhythmia due to their proven safety and efficacy [6, 7]. LLPs have advantages of avoiding complications encountered with conventional pacemakers (CPM) including infection, lead malfunction, and tricuspid valve regurgitation [8, 9]. Epicardial pacemaker is the standard recommendation in the setting of prior tricuspid valve surgery. However, prior cardiac surgeries are usually associated with significant pericardial adhesion, and most surgeons prefer not to implant epicardial leads in this setting due to impaired electrical properties of pericardial leads in the setting of pericardial adhesions. Therefore, LLPs can be a safe choice for patients with TV surgeries and postoperative AVB. There is few data about the LLP implantation in the presence of the bioprosthetic TV (BTV) [10, 11, 12]. In this report, we described a case of Micra‐VR implantation across the BTV in a patient with repaired congenital heart disease.

## 2 Case History

A 21‐year‐old man, known case of Pulmonary Valve (PV) Atresia, Large Atrial Septal Defect (ASD), and Patent Ductus Arteriosus (PDA) who underwent pulmonary valvotomy and PDA closure shortly after his birth, presented with exacerbation of dyspnea and peripheral edema. Right heart catheterization and transesophageal echocardiography revealed moderate LV dysfunction (ejection fraction :35%), severe right atrial(RA) enlargement (RA volume index:68CC/m^2^), moderate right ventricular (RV) enlargement (RV internal diameter = 3.8 cm), moderate to severe RV dysfunction (RV tricuspid annular plane systolic excursion (TAPSE):16 mm, RV S′ by Tissue Doppler Imaging :8 cm/s), severe pulmonary insufficiency (pulmonary pressure half‐time [PHT]:70 milliseconds), severe secondary tricuspid regurgitation (due to large ASD and RV enlargement), and large secundum ASD (size: 1.7 cm × 1.3 cm) with significant bidirectional shunt. He underwent simultaneous bioprosthetic replacement of PV (Perimount 25), tricuspid valve (Magna Ease 31), and ASD closure.

One week after surgery, he became bradycardic, and electrocardiogram showed complete AVB. Considering the persistence of AVB for more than a week, it was decided to implant a permanent pacemaker. As he had undergone recent BTV replacement, insertion of CPM was not preferred (due to increase risk of valve dysfunction and developing infection by CPM) [8]. So, the options were placement of epicardial pacemaker, coronary sinus (CS) lead, or a LLP. As the patient had undergone multiple cardiac surgery with resultant pericardial adhesion, cardiac surgeon refused to implant an epicardial lead. Implantation of a CS lead was impossible due to the absence of proper cardiac vein. Finally, it was decided to implant a LLP (Micra, Medtronic Inc).

## 3 Methods

The procedure was performed according to the standard technique; first we implanted the LLP in the apicoseptal area, however, electrical measures were not acceptable. Acceptable position was obtained in mid‐RV septum. Electrical measurements showed R wave amplitude of 10 mV, pacing impedance of 830 Ω, and pacing threshold of 1.0 V @ 0.24 ms. Pull and hold test was acceptable. Finally, tether was cut; delivery and introducer sheath were removed; and access site was closed using figure‐of‐eight suture. Patient was transferred to ward with good and stable condition.

**FIGURE 1:**
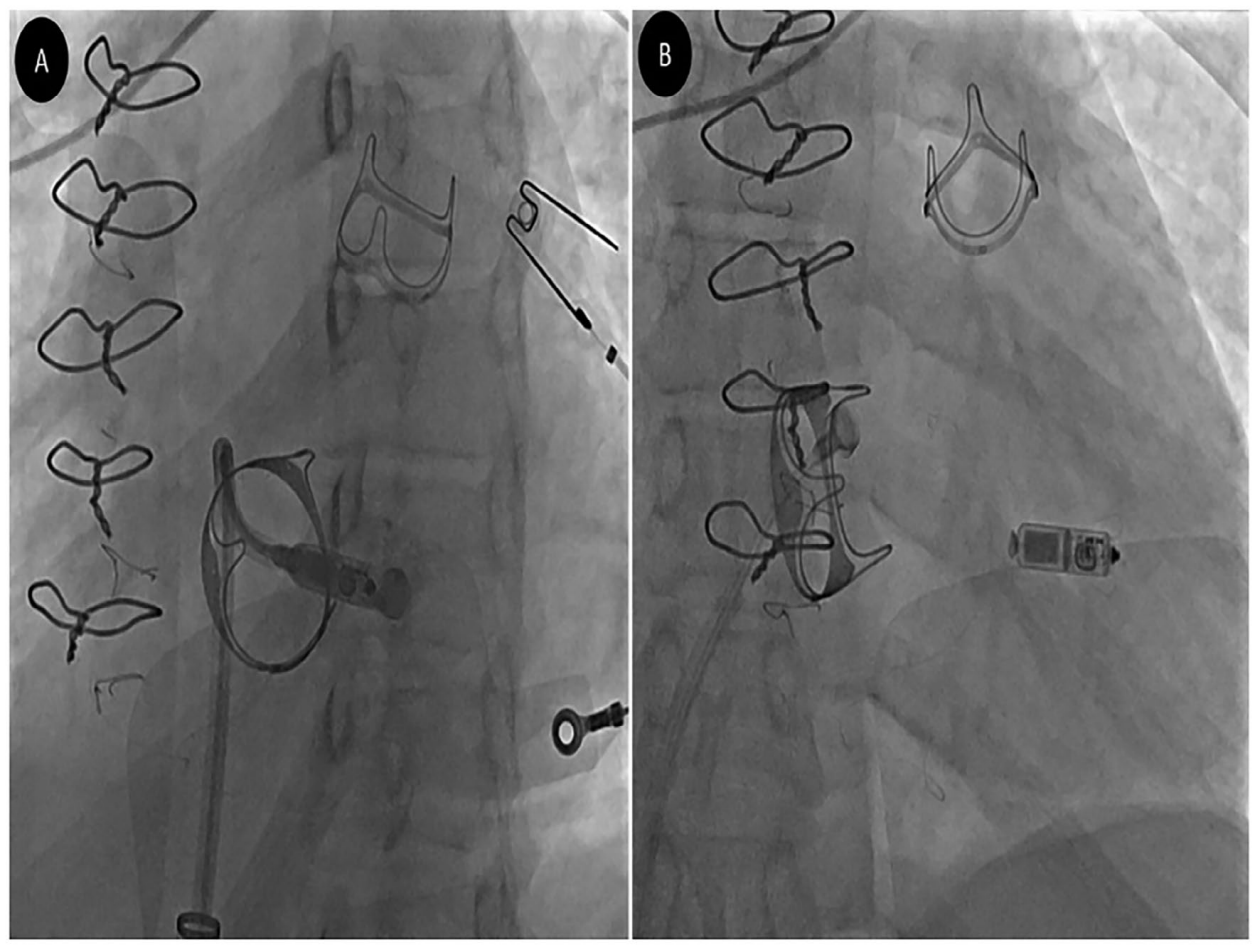
(A) Left anterior oblique (LAO) and (B) right anterior oblique (RAO) views after Micra deployment. LAO view is helpful to visualize the tricuspid annulus as a clock to be crossed exactly in the center and helps to confirm septal orientation of Micra before deployment. RAO view is used to establish the correct advancement of the Micra delivery system across the tricuspid valve and to evaluate the proper distance of implantation site from the valve.

Fluoroscopic oblique views were essential for a correct engagement of the tricuspid ring without injuries to the BTV. Left anterior oblique (LAO) view 40° was helpful to visualize the tricuspid annulus as a clock to be crossed exactly in the center. Right anterior oblique (RAO) view 30° was used to establish the correct advancement of the Micra delivery system across the tricuspid valve and to evaluate the proper distance of implantation site from the valve (Figure 1).

## 4 Results

**FIGURE 2:**
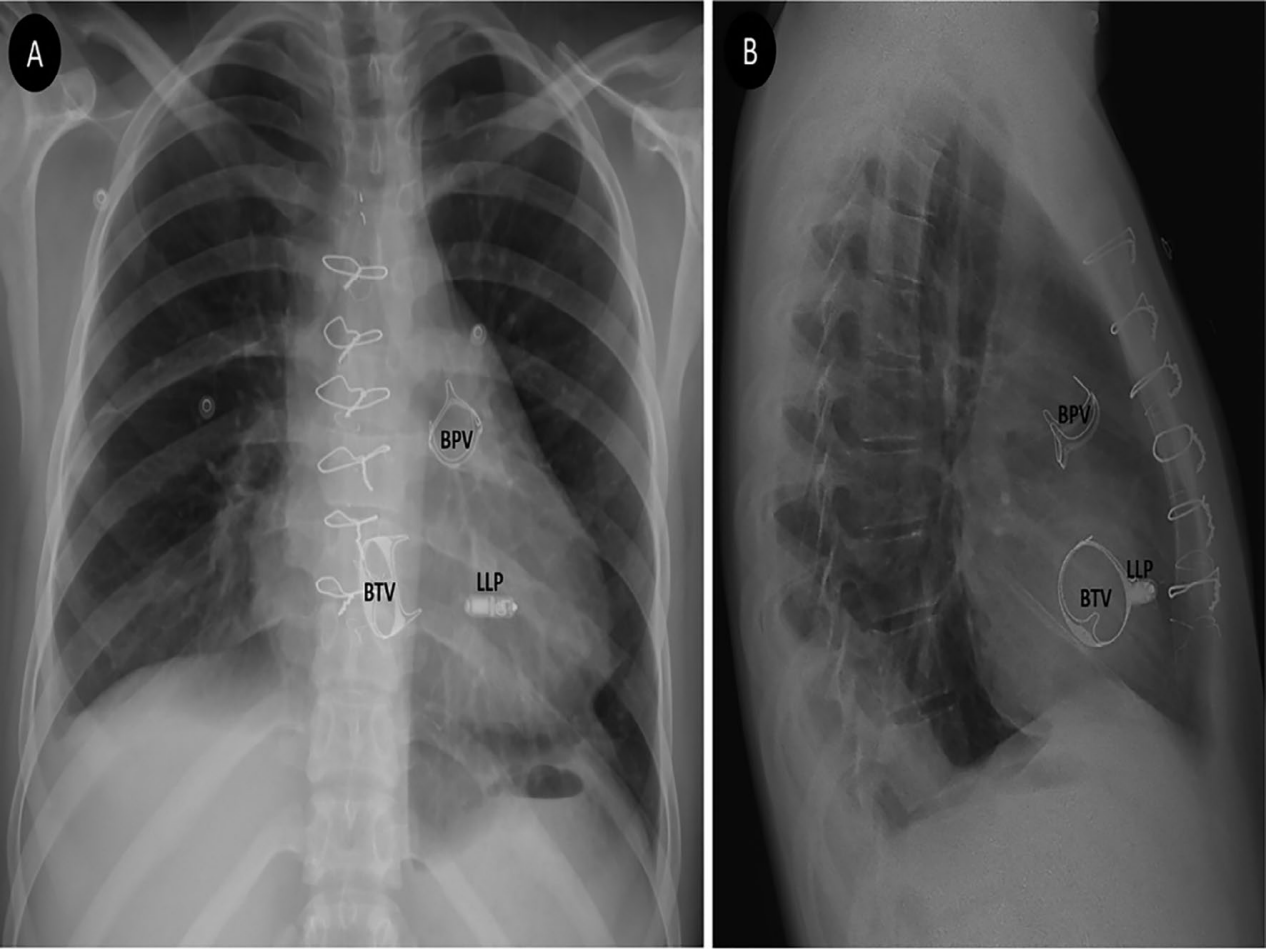
(A) Posteroanterior and (B) lateral chest radiographic views the day after implant. Bioprosthetic tricuspid valve (BTV), bioprosthetic pulmonary valve (BPV), and leadless pacemaker (LLP) are clearly shown in each view.

The day after the implantation, interrogation of the Micra‐VR revealed satisfactory parameters with a sensed R wave of 11.4 mV, the impedance of 820 Ω, and threshold of 0.63 V @ 0.24 ms. Chest radiography showed proper Micra location in the mid‐RV septum (Figure 2). Transthoracic echocardiography showed no pericardial effusion. During 7‐month follow‐up, the patient was asymptomatic and free of any complications.

## 5 Discussion

In this report, we presented successful Micra implantation through BTV in a patient with repaired congenital heart disease. The procedure was straightforward without any complications. During follow‐up, the patient was asymptomatic and the Micra interrogation showed proper functioning.

Tricuspid valve surgery carries a significant risk of conduction disorders requiring PPM implantation. The implantation rate decreased over time from 13% to 22% before 2000 [13] to 5–11– in the recent years [14]^.^ The PPM implantation after TV surgery involves technical challenges that must be acknowledged by the implanters to select the best technical option in each patient.

Several approaches have been reported: epicardial leads, CPM, His‐bundle pacing, leadless pacing, or coronary sinus leads [15].

1. Although epicardial PPMs are proven to provide adequate pacing, the reliability of endocardial leads has been shown to be superior to the epicardial systems [16]. This is especially applicable if patients had previous multiple cardiac surgeries with resultant pericardial adhesion, since surgeons may have a tough time to find a ventricular site with acceptable pacing thresholds.
2. CPM can interfere with the function of tricuspid valve, leading to significant morbidity and mortality rates through hemodynamic impairment. The presence of transvenous lead was an independent predictor of tricuspid regurgitation (TR) during follow‐up [17]. Although there is no clear evidence of increased TR after lead implantation in the presence of BTV, most operators prefer to avoid transvenous lead in these patients.
3. His‐bundle pacing (HBP) is a more physiologic form of pacing compared to ventricular pacing. This could be an interesting alternative for treating AVBs after TV surgeries, especially as the block site is nodal in most cases. HBP has been described to be feasible in small series (*n* = 10) of patients after TV repair but none with TV replacement [18]. In these settings, the TV ring may act as a radiographic marker of the his‐bundle and facilitate the implantation.
4. Since Cardiac Resynchronization Therapy (CRT) emerged as a cornerstone treatment for advanced heart failure patients, rare data have been published in the literature regarding CS pacing after TV surgery. Only one small series of 17 patients (11 TV repairs and 6 TV replacements) was published [19]. Due to the right atrial dilatation and resulting malposition of the CS ostium, CS catheterization and lead placement may be more challenging in this specific situation compared to typical CRT patients.
5. There are currently no large data about the safety and efficacy of leadless pacemakers in patients after TV surgery, current few studies support implanting LLPs in patients with bioprosthetic TV due to less major complications such as lead infection or valve dysfunction [10, 12, 15, 20, 21, 22].

LLP is associated with fewer infectious and lead‐related or pocket‐related complications in compare to CPM [8, 23].

The Micra Transcatheter Pacing Study, a multi‐site, single‐arm clinical trial conducted at nearly 70 centers around the world, assess safety and efficacy of Micra Pacemaker. The study has two primary endpoints: (i) a safety endpoint assessing freedom from major complications and (ii) effectiveness endpoint to evaluate pacing capture thresholds.

According to the results of this trial, Micra patients free from major complications (death, hospitalization, permanent loss of device function, and system revision) are significantly higher than 83% and the percentage of Micra patients with both low and stable thresholds is significantly higher than 80% [20].

Afzal and his colleagues demonstrated on a multicenter experience that implanting Leadless Pacemakers Across Bioprosthetic and Repaired TV is a safe and feasible option without any significant major complications [21].

In another study, a total of 14 patients underwent LLP implantation early after TV surgery. No procedure or device‐related complications happened during or after implantation and the procedure does not affect TV or bioprosthesis function in transthoracic echocardiography. So implantation of an LLP early after TV surgery is a safe option [22].

A retrospective review on complications of LLPs versus CPMs showed that LLPs appear to have a better safety profile than CPMs. There was a low pocket site and lead‐related infections in LLP as compared to CPM. However, LLPs can have twice the risk of pericardial effusion than CPMs, but this was not statistically significant [24]. Thus LLP implantation is an emerging technology validated in clinical studies and real‐world setting with the potential advantage of overcoming some of the limits of the conventional pacing lead such pocket infection. Micra LLP do not need extraction after battery depletion because LLP is endothelialized into ventricle and according to the existing studies, up to 3 LLPs (with battery longevity of 10–12 years) can be placed inside the RV. Therefore, there is no need to remove the previous LLP, and a new one can be implanted into the RV [23], so it prevents further open surgeries and the risk of post operation complications. LLP implantation after BTV might represent an ideal option in this setting by eliminating the risks related to the lead's presence across the bioprosthetic valve, including valve dysfunction and valvular endocarditis [8, 25, 26].

- Thus LLP implantation is an emerging technology validated in clinical studies and real‐world setting with the potential advantage of overcoming some of the limits of the conventional pacing lead such as need for extraction after battery depletion. LLPs overcome this limit and do not need extraction after battery depletion because LLP is endothelialized into ventricle and according to the existing studies, up to 3 LLPs (with battery longevity of 10–12 years) can be placed inside the RV. Therefore, there is no need to remove the previous LLP, and a new one can be implanted into the RV [23], so it prevents further open surgeries and the risk of post operation complications. LLP implantation after BTV might represent an ideal option in this setting by eliminating the risks related to the lead's presence across the bioprosthetic valve, including valve dysfunction and valvular endocarditis [8, 25, 26].

## Author Contributions

**Majid Haghjoo:** conceptualization, data curation, supervision, writing – review and editing. **Mahsa Mohammadi:** conceptualization, data curation, supervision, writing – original draft, writing – review and editing. **Mohammadreza Iranian, Amirfarjam Fazelifar**, and **Sedigheh Saedi:** data curation; writing – review and editing. **Yaser Toloueitabar:** data curation; writing – original draft, writing – review and editing.

## Consent

Written informed consent was obtained from the patient to publish this report in accordance with the patient consent policy of the Clinical Case Reports journal.

## Data Availability

The authors have nothing to report.
